# The Roles of Fatty Acids and Apolipoproteins in the Kidneys

**DOI:** 10.3390/metabo12050462

**Published:** 2022-05-20

**Authors:** Xiaoyue Pan

**Affiliations:** 1 Department of Foundations of Medicine, New York University Long Island School of Medicine, Mineola, New York, NY 11501, USA; xiaoyue.pan@nyulangone.org; 2 Diabetes and Obesity Research Center, NYU Langone Hospital—Long Island, Mineola, New York, NY 11501, USA

**Keywords:** apolipoprotein, fatty acid, chronic kidney disease, renal lipotoxicity, proximal tubule epithelial cells

## Abstract

The kidneys are organs that require energy from the metabolism of fatty acids and glucose; several studies have shown that the kidneys are metabolically active tissues with an estimated energy requirement similar to that of the heart. The kidneys may regulate the normal and pathological function of circulating lipids in the body, and their glomerular filtration barrier prevents large molecules or large lipoprotein particles from being filtered into pre-urine. Given the permeable nature of the kidneys, renal lipid metabolism plays an important role in affecting the rest of the body and the kidneys. Lipid metabolism in the kidneys is important because of the exchange of free fatty acids and apolipoproteins from the peripheral circulation. Apolipoproteins have important roles in the transport and metabolism of lipids within the glomeruli and renal tubules. Indeed, evidence indicates that apolipoproteins have multiple functions in regulating lipid import, transport, synthesis, storage, oxidation and export, and they are important for normal physiological function. Apolipoproteins are also risk factors for several renal diseases; for example, apolipoprotein L polymorphisms induce kidney diseases. Furthermore, renal apolipoprotein gene expression is substantially regulated under various physiological and disease conditions. This review is aimed at describing recent clinical and basic studies on the major roles and functions of apolipoproteins in the kidneys.

## 1. Introduction

Chronic kidney disease (CKD) is the underlying cause of kidney dysfunction; early observations that fatty acid is a key component of renal lipotoxicity gave rise to the fatty acid hypothesis for the pathogenesis of CKD. CKD is closely associated with a multitude of metabolic diseases, including obesity, insulin resistance, type 2 diabetes, hypertension, dyslipidemia and atherosclerosis [1,2,3,4,5,6]. Nearly 800,000 people in the United States have end-stage renal disease, according to kidney disease statistics for 2022. CKD is a major health challenge worldwide. If human CKD is identified early, medications and changes in lifestyle and environmental factors can rescue kidney function [7]. Increases in cardiovascular disease (CVD) strongly correlate with the development of CKD and circulating lipid levels [8]. CKD is also a risk factor for CVD. Advanced stages of CKD are associated with cardiovascular risk manifesting as coronary artery disease [9,10]. Obesity, diabetes, age and sleep disorders are common risk factors contributing to the progression of CVD and CKD [4].

Plasma lipid composition and apolipoproteins are a composition of lipoprotein. Plasma lipoproteins include chylomicrons, chylomicron remnants, very-low-density lipoproteins (VLDL), intermediate-density lipoproteins (IDL), low-density lipoproteins (LDL), high-density lipoproteins (HDL) and Lipoprotein(a) (Lp(a)). Lipids metabolism and transport and lipoprotein metabolism and particles are crucially essential contributing factors to CVD. Dyslipidemia and lipoprotein abnormalities are established CVD risk factors and are also common in patients with all stages of CKD. Early CKD is characterized by low levels of HDL, high levels of triglycerides and normal or elevated levels of LDL cholesterol [11,12]. Advanced CKD does not result in significant changes in LDL cholesterol levels, thus suggesting that LDL cholesterol is not a clear CVD risk factor in advanced CKD. Low cholesterol levels in the plasma in end-stage renal disease (ESRD) are associated with high mortality risk [13,14,15], possibly because of chronic inflammation and malnutrition; this result apparently contradicts the established relationship between higher lipid levels and atherosclerosis in the general population. However, high levels of the lipoprotein Lp(a) in CKD are strongly associated with atherosclerosis [16]. Lp(a) is an LDL-like particle linked to apolipoprotein B (apoB)-100 through a single disulfide bond [17]. Plasma Lp(a) in kidney disease is regulated by the glomerular filtration rate (GFR) and is induced in the earliest stages of renal impairment [18]. In addition, advanced CKD is associated with high triglyceride levels and triglyceride-rich and apoB-containing chylomicrons, very-low-density lipoproteins (VLDL) and intermediate-density lipoprotein particles [19,20]. Human and animal studies have shown that lipoprotein composition may affect CVD pathological procession and risk [10,14,21]. Lipoproteins and lipids are associated with the initiation and progression of CKD in animal models. However, whether lipoproteins and apolipoproteins prevent the development and progression of human renal disease is unknown, and many questions remain unanswered.

Patients with CKD show alterations in serum fatty acid levels and renal fatty acid metabolism disorders, thus resulting in mitochondrial dysfunction and cellular damage [21,22]. Increases in serum fatty acids are associated with not only effects on the heart but also the progression of kidney damage [23,24]. In recent years, many studies have highlighted the important role of lipid metabolism in the kidneys through mechanisms involving fatty acid and renal lesions leading to kidney dysfunction [21,23,25,26,27,28]. First, the kidneys take up fatty acids from the circulation [21,29]. Second, fatty acid synthesis and oxidation occur in the kidneys [28]. Third, and most importantly, fatty acids are assembled and secreted by the kidneys into the circulation [30,31].

**Figure 1:**
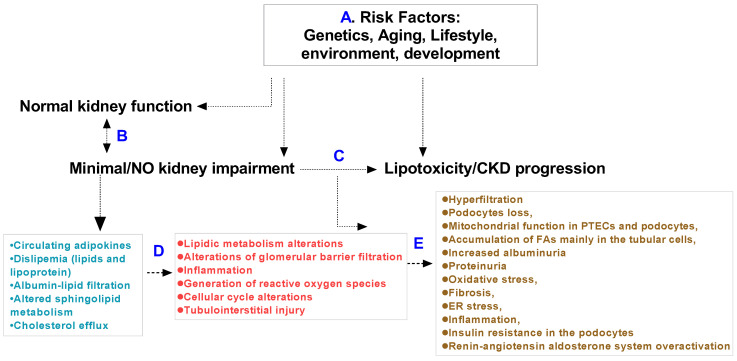
Mechanistic pathways of renal lipotoxicity and CKD: hypothetical pathways whereby disease risk factors (**A**) impose a requirement for constant adaptation (**B**) to maintain normal kidney function, and (**C**) minimal or no kidney impairment can cause lipotoxicity and progression of impairment and CKD according to the risk factors present. In these processes, many factors such as circulating adipokines, dyslipidemia, albumin-lipid filtration, altered sphingolipid metabolism and cholesterol efflux can cause early-stage renal steatosis through lipidic metabolism alterations, alterations in glomerular barrier filtration, inflammation, generation of reactive oxygen species, cell cycle alterations and tubulointerstitial injury (**D**), then develop mid-stage and end-stage CKD including in several factors (**E**) such as hyperfiltration, podocyte loss, mitochondrial function in proximal tubule epithelial cells (PTECs) and podocyte cells, accumulation of FAs mainly in the tubular cell. These different pathways may result in lipotoxicity and CKD progression.

Lipotoxicity can occur in the liver, skeletal muscle and heart. The accumulation of lipids in the tubular cells of kidneys are described as the main cause of lipotoxicity in many reviews. Fatty acids and renal proximal tubule epithelial cells contribute to kidney pathology [32,33,34]. According to observations in rodents, kidney dysfunction is related primarily to the circulation of lipids, and apolipoproteins also contribute to kidney tissue lipid-induced pathology [21,28,35,36,37]. Here, we summarize the fatty acids and apolipoprotein families in human or animal kidneys whose function, physiology and pathology result in a renal lipotoxicity phenotype and CKD. Age, sex, genetics, lifestyle, environment and anatomical and physiological development should be thoroughly considered in preclinical or normal kidney function in humans (Figure 1).

So far, several studies [14] have suggested that HDL deficiency and dysfunction, increased VLDL, IDL and triglyceride levels are important factors in CKD; decreased apoAI levels and an increased apoCIII/CII ratio are also important factors in CKD. CKD is associated with lipoprotein abnormalities, including normal to increased LDL levels and increased oxidized LDL levels. In addition, lipidomics studies [14] have suggested that (1) increased free fatty acids glycerolipid and glycerophospholipid levels are associated with CKD and (2) there is a negative relationship between the estimated GFR (eGFR) and methylhexadecanonic acid and 3-oxooctadecanoic acid, increased palmitic acid and monounsaturated acid levels and decreased polyunsaturated acid levels in CKD.

## 2. Physiological Roles of Fatty Acids in the Kidneys

In humans, triglyceride content differs between the kidneys and the liver. The total renal lipid content is approximately 3% of the kidney wet weight [38], and the total hepatic lipid content is approximately 4–5% of the liver wet weight [39], thus suggesting that the kidney triglyceride content is markedly different from that in the liver.

In the 1960s, studies established the renal lipid uptake in humans, measuring the lipid levels and comparing the differences in lipid concentrations between the feces and urine [40]. Early studies showed that the human kidneys contribute to systemic lipid metabolism, but this contribution is generally deemed insignificant in comparison to those of the intestines and liver [34]. The hypothetical mechanism is that re-absorptive endocytosis of filtered albumin determines the total influx of free fatty acids (FFAs) in the proximal tubules, thus indicating whether this process causes FFA accumulation in the kidneys and induces lipotoxicity. Studies have shown that the daily reabsorption of albumin by proximal tubules is approximately 5–50 µmol/day [41] Thus, 5–50 µmol FFAs might enter the proximal tubules each day, and 5% of FFAs can be delivered to the proximal tubules from the apical side through reabsorption [39]. The uptake from the basolateral side through circulation or synthesis in cells accounts for approximately 95% of the FFAs in proximal tubule cells, thus suggesting that the FFA uptake from the proximal tubule lumen through the apical side is less than the uptake from the circulation.

Renal FFAs are mainly taken up from the basolateral side, owing to high concentrations of FFAs in circulation [39]. A lack of cellular fatty acids can cause imbalances in apical fatty acid uptake through increasing albumin filtration and, consequently, FFA uptake from the apical side [42,43]. Apical and basolateral FFA uptake may result in distinct intracellular fates. Studies have also shown that albumin-bound FFAs induce macropinocytosis in podocytes, and renal FFAs induce angiopoietin-related protein 4 in podocytes and the circulation in the experimental model of minimal change disease (MCD) and human disease and may induce renal injury [44]. In addition, lipid synthesis from nonlipid substrates, such as carbohydrates, acetic acid and acetate, plays an important role in the normal kidneys. Renal expression of sterol regulatory element-binding proteins (SREBP)-1 has been found to regulate renal de novo lipogenesis [45,46]. Several studies have shown that the molecular mechanisms underlying renal FFA accumulation in the kidneys are due to [36,47,48]: (1) enhanced expression of SREBP-1c, Srebp2 and carbohydrate response element-binding protein (ChREBP); (2) decreased expression of peroxisome proliferator-activated receptor (PPAR)-alpha and -delta, which cause decreased fatty acid oxidation; and (3) decreased expression of nuclear receptor farnesoid X receptor (FXR) alpha and beta and small heterodimer partner (SHP). These transcription factors may play important roles in the increased expression of profibrotic growth hormones and proinflammatory cytokines and the elevated oxidative stress in the kidneys.

Under normal conditions, the kidneys take up FFAs from the circulation in fasted animals but add FFAs into the circulation in fed animals [31], thus suggesting that the transport of FFAs between the circulation and renal cells may be bidirectional. Fasting-induced lipid accumulation in the kidney cortex was demonstrated in tubule cells [31]. Thus, fasting-induced triglyceride accumulation may increase glomerular filtration and tubular re-uptake of albumin-bound fatty acids. Moreover, caloric restriction regulates age-associated renal disease partly through the modulation of renal SREBP expression and decreases renal lipid accumulation in proximal tubule epithelial cells in aged C57BL/6J mic [49].

## 3. Molecular and Pathological Roles of Fatty Acids in Renal Lipotoxicity and CKD

### 3.1. Molecular Mechanism of Fatty Acids in Renal Lipotoxicity

Lipids in circulation can accumulate in the kidneys, thus resulting in kidney lipotoxicity through fatty acid metabolic processes or the toxic effects of saturated fatty acids [22,35,39,50,51]. However, FFA accumulation and the induction of lipotoxicity have appeared as one of the major health problems around the world [14,21,28,34,35,39,47,48,52,53,54,55,56,57,58,59,60].

**Figure 2:**
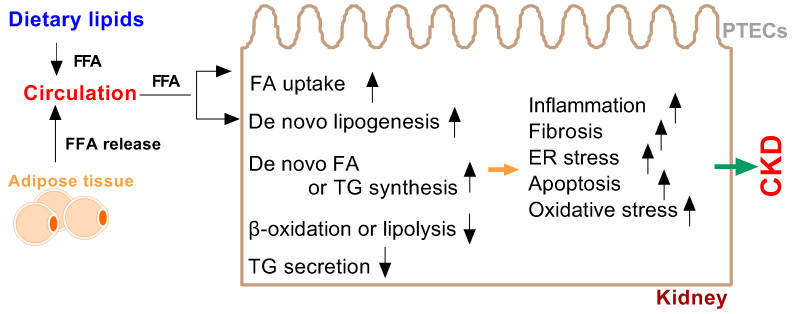
High free fatty acids (FFA) in circulation, originating from dietary lipids and adipose tissue, cause fatty acid metabolism disorders, thus acting as major risk factors for lipotoxicity and CKD in the kidneys. Increased fatty acid (FA) uptake increases de novo lipogenesis, increases de novo FA and TG synthesis, decreases β-oxidation or lipolysis and decreases triglyceride (TG) secretion, thus resulting in lipid accumulation in the PTECs of the kidneys. The lipotoxic effects caused by the ectopic accumulation of lipids in the kidneys include oxidative stress, fibrosis, endoplasmic reticulum (ER) stress, inflammation and other risk factors that cause lipotoxicity-induced CKD.

Renal lipotoxicity is associated with inflammation and fibrosis; it induces oxidative stress and albuminuria and regulates intra-cellular signaling pathways in renal lipid metabolism [39,61]. Kang et al. reported that incomplete fatty acid oxidation in renal tubular epithelial cells plays a key role in the development of renal fibrosis [62]. Several mechanisms are involved in the regulation of kidney function resulting in lipotoxicity. The adipose tissue releases lipids into the bloodstream and alters lipid signaling [4,61,63,64] (Figure 2).

First, mechanistic studies of renal lipotoxicity have shown increased lipogenesis and decreased lipolysis in endothelial cells, podocytes and proximal tubular epithelial cells [35,64,65]. Second, increases in Srebps (transcription factors associated with lipogenesis) cause the accumulation of fatty acids and exhaustion of mitochondrial β-oxidative capacity in the kidneys [36]. Third, downregulation of the nuclear receptor FXR, and decreased carbocilesterase-1 and lipolysis, induce the esterification of fatty acids with glycerol and the formation of lipid droplets, thereby inducing renal steatosis through the deregulation of adipocytokines and their functions in the kidneys [36,48]. Fourth, hypertriglyceridemia causes the accumulation of fatty acids in renal tissue, owing to increases in the cluster of differentiation 36 (CD36) protein and greater uptake of fatty acids, thus resulting in the formation of reactive oxygen species (ROS), which induce oxidative stress in the kidneys [66]. Fifth, lipotoxicity was shown to be involved in various cellular signaling pathways, including the increased transcription of PPAR-γ, activation of lipogenesis, decreased PPARα, activation of lipolysis and increased endoplasmic reticulum and lysosomal dysfunction from the recruitment of macrophages to adipose tissue and the formation of ROS^35^. Finally, activation of AMP-activated protein kinase (AMPK) is associated with high fatty acid β-oxidation and decreased apoptosis through systemic inflammation [67].

### 3.2. Glycotoxicity and Lipotoxicity

Lipotoxicity is characterized by the ectopic accumulation of lipids in tissues other than adipose tissue. In the obesity model, adipokines increase renal lipotoxicity, thereby inducing oxidative stress and inflammation, as well as stimulating renal sympathetic nervous activity [68]. Lipid droplets are observed in the renal cells of obese people [69]. Renal metabolic deregulation aggravates lipid deposition, thus leading to a decrease in energy expenditure that ultimately induces apoptosis and contributes to CKD [70]. The activation of PPARγ by PPARγ agonists is a lipolytic mechanism protective against kidney changes caused by obesity [71]. In addition, distinguishing the effects of glycotoxicity (toxicity from advanced glycation end products formed from excessive sugars) from lipotoxicity is difficult in clinical practice, mainly because of the long exposures and synergistic interrelationships among the mechanisms [65]. Moreover, differentiation between the focus on glucose as the cause of a condition (glucocentric mechanisms) and the focus on lipids as the cause of a condition (lipocentric mechanisms) may be confusing, given that both are involved in the progression of energy imbalance diseases and kidney disease [65]. Lipotoxicity is also associated with dysfunctional intracellular signaling [72] and an insulin resistance response in the kidneys [61,65].

### 3.3. DKD and CKD

Most patients with diabetes (40%) develop kidney disease and end-stage kidney disease (ESKD), the latter of which is characterized by immune cell infiltration, glomerular injury and tubulointerstitial damage [73] Current therapies do not induce remission in all patients, and many individuals progress to kidney failure. Recently, Mori et al. showed that kidney injury molecule-1 (KIM-1) is highly expressed in the proximal tubules and is elevated in the blood and urine in patients with diabetes [74]. They found that KLM-1 regulates palmitic acid–bound albumin uptake in the proximal tubules, thus causing tubule injury, DNA damage and proximal tubular cell-cycle arrest, and additionally inducing interstitial inflammation, fibrosis and glomerulosclerosis [74]. Sodium-glucose co-transporter-2 (SGLT2) inhibitors, a target of diabetic kidney disease (DKD) therapy, were also validated [5,75]. Mori et al. found that the small molecule Bcl-2/Mcl-1 inhibitor TW-37 also inhibits KIM-1-mediated palmitic acid-albumin uptake in a mouse kidney model [74]. The authors proposed a different strategy for therapeutically targeting the kidneys in patients with CKD using a KIM-1 inhibitor. SGLT2 inhibitors are approved for clinical use [76,77]. However, whether combined KIM-1 inhibitor and SGLT2 inhibitor therapy might decrease the risk of CKD or DKD remains unknown.

### 3.4. Different Single Cell Function and CKD

Endothelial cells are a therapeutic target against lipotoxicity at the systemic level. Although endothelial cells do not appear to be predisposed to lipid accumulation, they play an important role in the transport of lipids to other tissues, particularly in the kidney region [78,79]. Endothelial cells are the main source of the lipid supply to glomerular cells through the co-expression of vascular endothelial growth factor B (VEGF-B) and mitochondrial proteins [80]. Endothelial glycocalyx dysfunction, including renal cholesterol accumulation and modified lipoprotein accumulation, is present in patients with CKD [79]. Mesangial cells are specialized cells in the kidneys that present LDL receptor (LDLr) and CD36 expression. Increased accumulation of foam cells and the presence of intracellular lipid droplets in macrophages and/or foam cells of the kidneys are observed in focal segmental glomerulosclerosis (FSGS) and diabetic nephropathy [81]. Cytokines such as tumor necrosis factor (TNFα) and interleukin 1 beta (IL-1β) are shown to regulate LDLr-mediated cholesterol uptake through increasing SREBP translocation and stimulating foam cell formation in macrophages and mesangial cells in the kidneys [82,83,84].

## 4. Roles and Biological and Pathological Functions of Apolipoproteins in the Kidneys and CKD

Lipids and lipid transport-associated proteins are elevated in the urinary excretion of children with kidney stones and hypercalciuria [85]. However, the roles of apolipoproteins in kidney stone formation and the effects of dietary changes on lipoprotein-associated urinary excretion remain unclear and should be investigated further [86]. In addition, the molecular mechanisms underlying the roles of apolipoproteins in kidney diseases remain unclear.

Below, recent findings regarding changes in the most abundant apolipoproteins are discussed. Apolipoprotein dysfunction can damage kidney function and morphology, according to clinical studies. The gene expression of all apolipoproteins was detected in the kidneys (Table 1).

**ApoA-I**

ApoA-I is the main protein in HDL particles. It has a size of 28.1 kDa molecular weight (MW) and is catabolized in the liver and kidneys. ApoA-I is considered protective against CVD [87,88,89,90,91]. ApoA-I plays a critical role in the ATP binding cassette subfamily A member 1-dependent efflux of excess cholesterol and phospholipids from peripheral tissue in reverse cholesterol transport [87]. Studies showed that the dissociation of apoA-I from HDL or the failure of apoA-I to incorporate into HDL enhances renal apoA-I catabolism [92].

ApoA-I clearance is associated with the kidneys in rodents. The kidneys are major organs removing apoA-I from the body [93], and urinary apoA1 concentration is positively associated with renal dysfunction and renal disease over time [94]. Recently, Jacobs-Cacha et al. reported that apoA-I disorder is associated with recurrent focal segmental glomerulosclerosis [95]. A misprocessed form of apoA-I precursor was found in the urine in approximately 40% of patients with primary FSGS on a kidney transplant waitlist [95]. Recently, Saraf et al. showed that ApoA1 is a candidate gene in sickle cell disease-associated nephropathy [96]. The apoB/apoA1 ratio was shown to be associated with CVD and CKD. Zhao et al. showed that the serum apoB/apoA1 ratio is associated with the progression of diabetic kidney disease in 258 patients receiving renal replacement therapy [97].

ApoA-I mimetic peptides are an emerging class of therapeutic agents whose antioxidant/anti-inflammatory properties and reverse cholesterol transport (RCT) are used to treat atherosclerosis and inflammatory disorders [88]. Decreased serum apoA1 has been demonstrated in patients with renal dysfunction [98]. Studies have shown that ApoA1 mimetic peptides may ameliorate nephropathy in a mouse model of atherosclerosis, such as ApoE-deficient mice [99]. ApoA1 mimetic peptides have also been found to decrease renal tissue lipid accumulation in Ldlr-deficient, ApoE-deficient mice fed a Western diet as well as 5/6 nephrectomy rats in a CKD model [100,101]. These findings suggest that apoA-I may play important roles in cholesterol transport, and APOA1 mimetic peptides may be useful for treating kidney disease.

**ApoA-II**

ApoAII exists as a 17.4 kDa homodimer. Like apoA-I, apoA-II is a major protein element of HDL [102]. ApoA-II is present in the first segment of proximal tubules and adjacent to the glomerulus. Although the role of human APOAII remains unclear, APOAII has anti-atherogenic effects [103,104].

ApoAII is a marker linking dyslipidemia and the risk of kidney stones in humans and other animals [105]. Because of its small molecular size, ApoA-II, like other apolipoproteins, might undergo reabsorption in the renal tubules or might pass through the glomerular sieves and be excreted in the urine [106,107]. SNPs in the ApoA-II gene promoter are associated with insulin resistance, which is a risk factor for type 2 diabetes (T2D), diabetic kidney disease, CVD and nonalcoholic steatohepatitis [108]. ApoA-II is implicated in renal amyloidosis [109]. The mutation of human APOA2 is associated with systemic deposition disease, such as renal amyloidosis or cardiomyopathy with atrial fibrillation. High apoA-II concentrations in the plasma are associated with a lower risk of death in patients with CKD [110]. Abnormal HDL apoA-I and apoA-II kinetics in 1255 patients receiving hemodialysis indicated that lower levels of apoA-II are primarily due to a decreased rate of production in patients with ESRD receiving hemodialysis [110,111]. In obese individuals or those with T2D, apoA-II is more hydrophobic than apoA-I, thus displacing apoA-I from HDL particles [112]; however, the underlying mechanism remains unknown. Recently, Brown et al. showed that ApoA2 mutation was associated with renal amyloidosis in a 63-year-old man with ESRD [113].

**ApoA-IV**

ApoA-IV is a 46 kDa apolipoprotein in HDL particles, which serves as a serum biomarker for renal injury as well as diabetic kidney disease [114,115]. Increased APOA-IV is associated with renal disorders, specifically in mild and moderate renal failure [116]. ApoA-IV protein is expressed in kidney tubular cells in humans and other animals. Baseline plasma apoA-IV and triglyceride concentrations are higher, and HDL cholesterol levels are lower in patients with CKD than in unaffected individuals [117]. ApoA-IV is an early marker of renal impairment [108]. Recently, Perampalam et al. reported that the downregulation of Dp, Rb-like, E2F and MuvB (DREAM), a transcriptional repressor, enhances ApoA4 protein levels and causes systemic amyloidosis in the heart, spleen, liver and kidneys [118]. ApoA-IV is also associated with changes in insulin resistance [119], which in turn are associated with CKD. Recently, Lee et al. reported that ApoA4 expression is increased by treatment with TNFα via the activation of TNF receptor 2 and nuclear factor kappa B signaling in injured kidney tubular cells [120]. However, the function of ApoA-IV in renal tissue injury is not fully understood and requires further study.

**ApoA-V**

ApoA-V is a 39 kDa apolipoprotein [121] that is positively correlated with HDL cholesterol in patients with ESRD [122]. Studies have shown that plasma APOA-V is lower in patients with diabetic or nondiabetic ESRD than in healthy individuals [123]. Two polymorphisms in APOA5 (1131T>C [rs662799] and T1259C [rs2266788]) are involved in lipid metabolism and are significantly associated with CKD stages 3–5 [124,125]. APOA5 T1259C (rs226788) is significantly associated with blood triglyceride levels in renal dysfunction [124], thus suggesting that modulation of APOA5 to regulate blood triglyceride levels might be a key factor contributing to the development of CKD as well as T2D nephropathy. Recently, de Luis et al. reported that the minor C allele of the APOA5 gene (rs662799) is negatively associated with plasma triglyceride levels, insulin levels and homeostatic model assessment for insulin resistance after a hypocaloric diet with a Mediterranean pattern [126].

**ApoB**

ApoB has two main forms, APOB-48 and APOB-100, which are 210 kDa and 550 kDa, respectively, and are found in intermediate-density lipoprotein, LDL, VLDL and chylomicrons [127,128,129]. ApoB mRNA is expressed in mammalian kidneys.

Studies have found that the plasma apoB/A1 ratio, but not apoB level, is associated with CKD progression and immunoglobulin A nephropathy [130,131,132], although neither apoA1 nor apoB alone is associated with renal dysfunction in heart failure [133]. Higher apoB/apoA1 ratios are significantly associated with lower eGFR [131]. The preoperative apoB/apoA1 ratio is also a useful marker in improving current prognostic evaluation and treatment decisions for patients with metastatic renal cell carcinoma [134]. Hypertriglyceridemia or hyper-apoB is associated with the highest risk of albuminuria [135,136]. ApoB-containing lipoproteins have been found to be important causes of elevated urinary albumin excretion rates in a study of 275 patient [135,136]. Increased serum APOB is associated with an elevated risk of a need for renal replacement therapy in patients with diabetic kidney disease [97]. The deposition of ApoB results in the progression of glomerulosclerosis [137]. Elevated plasma APOB is correlated with microalbuminuria and the development of overt nephropathy in T2D [97,138]. Ma et al. reported that polymorphisms in APOB are associated with diabetic kidney disease in Chinese patients with T2D [139]. Additionally, Kwon et al. also showed that high plasma APOB concentrations are associated with a higher risk of ESRD in 9403 participants [130]. In ESRD, owing to renal lipid supplies being overstepped for energy consumption, less fatty acid β-oxidation occurs; therefore, apoB-containing lipoproteins remove excessive triglycerides from the tubular epithelium [140]. These studies have suggested that ApoB is a risk factor for ESRD [130]. However, the underlying molecular mechanism remains unknown.

Several studies have indicated that lipoproteins might be produced by tubular epithelial cells rather than glomerular or vascular cells [22,26,64]. ApoB is produced mainly by tubular epithelial cells [140]. ApoB-containing lipoproteins in the kidneys may depend on lipid availability, which may be higher in the proximal than the more distal tubule cells of the kidney. In addition, mouse kidneys secrete apoB-containing lipoproteins [140,141]. ApoB-antisense locked nucleic acid oligonucleotides have been found to decrease ApoB expression by 90% in the renal cortex in wild type mice in vivo [142]. The repression of ApoB expression enhances fasting-induced triglyceride accumulation in the kidneys [31]. In mammals, kidney secreted apoB-100-containing lipoproteins decrease the accumulation of triglycerides in proximal tubule cells [143]. However, how apoB-containing lipoproteins are secreted from the kidneys remains unknown. ApoB-containing lipoproteins have been suggested to be produced by tubular epithelial cells rather than glomerular or vascular cells [140,143].

According to the two-point hypothesis, the first point is due to renal uptake of lipids (including phospholipids, albumin-bound FFAs and lipophilic vitamins), thus leading to the secretion of lipids into the circulation [39]. The second point is due to apoB secretion being the main pathway for assembly and resecretion of lipids from renal proximal tubule epithelial cells back into the circulation [130]. However, this hypothesis remains to be tested.

**ApoC**

The ApoC family has three members: ApoC-I, ApoC-II and ApoC-III. These low molecular weight apolipoproteins are components of chylomicrons, VLDL and HDL [33,144]. Like other low molecular weight apolipoproteins, ApoC-I and ApoC-II can diffuse via adsorption on dialysis membranes. The ApoC1–3 gene family might regulate lipoprotein metabolism in patients receiving hemodialysis [145].

**ApoC-I**

ApoC-I is a 7.6 kDa low molecular weight protein. High plasma apoC-I decreases the uptake of triglyceride-rich lipoproteins via hepatic receptors, such as the LDL receptor-related protein [146]. Plasma apoC-I also activates lecithin cholesterol acyltransferase (LCAT), thus inhibiting plasma phospholipase A2, cholesteryl ester transfer protein (CETP), lipoprotein lipase (LPL) and hepatic lipase, and playing important roles in lipid metabolism. Studies have shown that diminished ApoC-I might decrease LPL activity and the accumulation of triglyceride-rich lipoproteins that cause chronic renal failure [147,148]. This finding may be explained by the higher apoC-I clearance and, consequently, lower renal triglyceride accumulation in patients receiving dialysis than those not receiving dialysis [149]. Bus et al. found that polymorphisms in human APOC1 as well as mouse apoC1 increase the number of glomerular M1 macrophages [150] and are associated with the development of diabetic nephropathy in human apoC1 transgenic mice [145,150]. These data suggest that apoC-I plays an important role in the pathogenesis of glomerulosclerosis in nephropathy. Together, the findings show that apoC-I is involved in atherosclerosis and might cause the formation of glomerular nodules, which induce vascular damage [151]. Recently, Cui et al. showed that renal cancer samples display the induction of apoC1 expression; moreover, high levels of APOC1 are associated with poor survival times in clear cell renal cell carcinoma (ccRCC) [152]. ApoC-I is a novel pro-metastatic factor, and exosomes containing APOC-I are transferred from ccRCC cells to vascular endothelial cells [152]. This process activates the signal transducer and activator of transcription 3, thus promoting metastasis of ccRCC cells, according to in vitro and in vivo studies [153].

**ApoC-II**

ApoC-II is an 8.9 kDa protein that acts as a physiological activator of LPL and plays an important role in the efficient lipolysis of triglyceride-rich lipoproteins in circulation [154]. The progression of renal insufficiency is associated with marked increases in the triglyceride content of VLDL, LDL and HDL and is associated with high apoC-II and apoC-III levels in the plasma [155].

**ApoC-III**

ApoC-III is an 8.7 kDa protein. Like apoA-I in the kidneys, renal ApoC-III dysfunction is associated with renal insufficiency in T2D but does not affect albuminuria [60]. ApoC-III plays an important role in triglyceride transport and triglyceride homeostasis [155]. The concentration of ApoC-III in urinary excretion is significantly associated with urinary calcium excretion in children [156], thus suggesting that abnormalities in lipid metabolism and APOC3 might play a role in kidney stone formation [156]. However, ApoC’s physiological role in the kidneys remains to be established.

**ApoD**

ApoD, a 25–30 kDa protein cloned in 1980 [157], is a human-protein component of plasma HDL and is associated with lecithin-cholesterol acyltransferase and progesterone binding [158]. ApoD is expressed in the kidneys as well as the intestines, liver, central nervous system, testis and adrenal glands. ApoD is a lipid-transport protein that has been found in urine [159]. APOD is associated with renal function in African American participants in a hypertension Genetic Epidemiology Network [160]. ApoD may be associated with kidney failure (including creatinine, eGFR and urea). However, little is known regarding the function of this apolipoprotein in the kidneys.

**ApoE**

ApoE is a 34 kDa protein synthesized in the liver and kidneys [161]. The role of APOE in kidney pathogenesis has not been well studied, although APOE variants are associated with nondiabetic ESRD [162]. Several studies indicated that ApoE deficiency is associated with occasional glomerular capillary thrombosis and substantial glomerular and tubulointerstitial macrophage and lymphocyte accumulation [100,163,164]. APOE mutants lead to lipoprotein glomerulopathy, which is abnormal lipoprotein deposition in glomerular capillaries and mesangial proliferation, thus causing nephrotic syndrome [165,166]. In 5/6 nephrectomy ApoE-deficient mice, kidney function is diminished, and aortic plaques increase 6- to 10-fold, thus causing cholesteryl ester accumulation in foam cells [167].

In addition, ApoE2 homozygotes show glomerulopathy and lipoprotein thrombi [168]. ApoE2 has been associated with diabetic nephropathy with abnormal lipid metabolism. Serum apoE2 levels are associated with the severity of IgA nephropathy, and the apoE2 allele may play an important role in the progression to ESRD [169]. Like apoE2, apoE5 is a risk factor underlying lipid-induced kidney diseases 169].

**ApoF**

ApoF is a minor apolipoprotein in plasma LDL with a size of 35.3 kDa. ApoF is also called lipid transfer inhibitor protein (LTIP). Hepatic apoF negatively regulates plasma LDL levels and increases RCT in fat-fed hamsters [170]. ApoF inhibits CETP activity, thus increasing HDL cholesterol. ApoF may prevent atherosclerosis risk [170]. The regulation of APOF includes signaling receptor binding and lipid transporter activity [171,172]. ApoF is expressed in human kidneys. According to RNA-seq, the ApoF expression in the kidneys is one-third that in the liver. Aberrant LTIP activity has been shown in patients with uremia undergoing continuous ambulatory peritoneal dialysis [173]. However, the role of ApoF in the kidneys is unknown.

**ApoH**

ApoH is a 50 kDa protein present in the plasma, in free form and in combination with HDL. APOH mRNA and protein are mainly expressed in the proximal tubules in the kidneys [174]. ApoH is filtered by the glomeruli and then reabsorbed into renal epithelial cells [175]. High urinary concentrations of APOH have been observed in patients with Fanconi syndrome [176]. Genome-wide association studies have emphasized that APOH may serve as a novel locus modulating lipoprotein (a) levels in individuals of European ancestry [177]. To date, little is known regarding the role of ApoH play in kidney diseases.

**ApoJ**

ApoJ, also called clusterin, is a 75–80 kDa secretory glycoprotein with two 40 kDa heterodimeric protein forms. ApoJ is an HDL apolipoprotein [178,179] that is expressed in epithelial cells [180] and is associated with various disease states such as polycystic kidney disease, ischemic renal tissues and lupus-like nephritis [181], as well as several forms of acute and chronic renal disease [182,183,184]. ApoJ/clusterin-deficient aged mice showed a 75% increase in glomeruli in the kidneys and induced renal lipid accumulation [182,185,186].

**ApoL**

ApoL-I is 43 kDa in molecular weight. The apolipoprotein L gene family includes ApoL1–6 [187]. APOL-I and APOL-II co-localize with ApoA-I in HDL particles and play important roles in lipid exchange, transport and movement in the kidneys [188,189]. APOL-I protein is expressed in podocytes of the glomerulus, the proximal tubules and extra glomerular arterial endothelium in normal human kidneys [190]. APOL-I, which binds APOA-I, may modulate cholesterol efflux in podocytes under physiological conditions [191,192]. APOL-I is strongly associated with CVD and CKD [193]. ApoL-I dysfunction induces the pathogenesis of glomerular diseases such as HIV-associated nephropathy and FSGS [189]. Additionally, APOL1 is associated with recurrent FSGS after transplantation [194]. Recently, Zee et al. showed that glomerular APOL1 expression or APOL1 risk alleles are associated with cellular/tissue changes in patients with FSGS [195,196]. ApoL2 is found mainly in the brain [197], but its function remains unknown in the kidneys. ApoL3 and ApoL4 are associated with cholesterol and sphingolipid transport/recycling to the plasma membrane in the lungs and other tissues [190], but their functions are also unknown in the kidneys.

**ApoM**

ApoM is a small apolipoprotein of 26 kDa, which is highly produced in the liver and kidneys [198,199]; 95% of plasma apoM is associated with HDL, and 5% of plasma apoM is present in LDL, VLDL and chylomicron particles in humans and other animals [200]. ApoM has been reported to bind megalin and to be strongly expressed in kidney proximal tubular cells [201]. ApoM knockout mice show induction of apoptosis via mitochondrial and endoplasmic reticulum stress in renal tissue [202]. Studies have shown that ApoM functions as a natural carrier of sphingosine-1-phosphate (S1P), and ApoM and SIP are regulated by several transcription factors [203,204]. However, Brinck et al. reported elevated S1P and diminished apoM in HDL particles in patients with CKD [205]. Uremia increases plasma apoM by 25% but has no effect on S1P [198]. Svarrer et al. reported that urinary apoM serves as a biomarker of acute kidney injury in young children after heart surgery [206]. Plasma apoM is also associated with apoA-I-containing HDL and plays a key role in the biology of plasma HDL [198].

### Pathological Role of Apolipoproteins in Kidney Diseases

Apolipoproteins are composed of lipoproteins, which may play a key role in renal toxicity. Dysfunctional apolipoproteins induce glomerular and tubular damage [60]. Further exploration is needed to understand the relationship between apolipoproteins and renal injury. The regulation and possible roles of apolipoproteins in the kidneys, considering the specific physiology and pathophysiology of the kidneys, are summarized in Table 2. Hence, apolipoprotein dysfunction is associated with nephrotic syndrome with or without developing CKD. In turn, renal dysfunction is also associated with many disorders in lipoprotein metabolism leading to dyslipidemia and apolipoprotein dysfunction.

For example, plasma apoAI, apoAII nonB and ApoCIII nonB in HDL particles are negatively associated with LDL cholesterol in patients after renal transplantation [207]. ApoE deficiency has been shown to alter the plasma cholesterol concentration in lipoproteins in atherosclerosis and CKD [208,209,210]. Plasma apoB100 is significantly elevated during CVD and CKD, owing to the effect of apoB-100 on oxidized LDL-induced kidney cytotoxicity [211]. ApoA-IV is a possible link between lipoprotein function and the development of nephrotic syndrome with or without CKD, ApoA-IV accumulation in proximal and distal tubular cells may influence kidney cell recovery [212]. ApoL-I is considered to have an important function in the pathogenesis of CKD-induced diabetes [213]. APOL-I is also associated with an elevated risk of hypertensive disease, lupus and HIV-associated kidney disease [214]. The presence of two apolipoprotein L renal risk variants is rapidly emerging as a pathological mechanism that increases the risk of kidney disease [214,215].

Therefore, understanding the mechanisms associated with apolipoprotein-associated kidney disease is essential for successful treatment strategies that ameliorate fatty acid accumulation and apolipoprotein function in kidney cells and improve kidney metabolic health.

Apolipoproteins such as ApoB, ApoA-I, ApoE and ApoCs also play important roles in the viral pathogenesis and regulation of hepatitis C viral (HCV) entry, assembly and transmission [216]. HCV is inversely associated with the development of diabetes mellitus and CVD as well as CKD [217].

## 5. Future Perspectives and Conclusions

Obesity, diabetes mellitus and hyperlipidemia causing aberrant lipotoxicity are well-known pathological hallmarks of CK [64]. In recent years, increased attention has been paid to the accumulation of lipids through cell and tissue crosstalk in these renal pathologies. Aberrant lipid and apolipoprotein metabolism is associated with multiple kidney disease characteristics, such as increased renal inflammation and ROS production [39]. Therefore, maintaining kidney lipid accumulation is considered increasingly important in CKD.

Although many apolipoproteins are not well studied in the context of CKD, they are an interesting field of research because of their strong association with dysfunctional fatty acid and apolipoprotein metabolism. Substantial direct evidence that apolipoproteins are an important mechanism in the pathology of CKD is lacking for two main reasons. First, the abnormal accumulation of lipids in the kidneys has long been known, but the regulation of apolipoprotein function has only recently been reported, and increasing evidence indicates the importance of lipid metabolism in the kidneys. Second, apolipoproteins often stood in the shadow of general lipoproteins and are associated with heart and liver diseases in humans and other animal models; for example, the circadian clock was found to regulate apoB-containing lipoproteins, thereby Clock genes controlling plasma lipids and atherosclerosis [218,219]. Recently, the circadian clock gene Bmal1 was found to regulate lipoprotein assembly and secretion, thus controlling liver metabolism through ApoA4 in the liver [220]. However, how circadian clock genes regulate kidney apolipoproteins remains unknown. Only in the past few years have apolipoproteins been considered important elements of kidney lipoproteins; for example, apoL1 is an independent risk factor associated with CKD. These findings were based mainly on CKD genome-wide association studies revealing the apolipoproteins and possible apolipoprotein-related factors in the kidneys. To date, several studies have provided insight into how apolipoproteins are associated with CVD. Validation of these findings in the kidneys and in the context of CKD is needed, given the known heterogeneity in apolipoproteins across various tissues, cell types and the plasma [37,137,215]. Another overlooked aspect is whether the apolipoproteins in circulating lipoproteins might affect fatty acid functions in the kidneys. This possibility is worthy of study, particularly given that the renal core lipid, surface lipid and apolipoprotein composition analysis of lipoprotein particles, cell motility and spatial heterogeneity in apolipoprotein function and fatty acid metabolism in the kidneys, and that the lipoprotein size, intensity and lipid composition and apolipoprotein dispersal observed through kidney lipid staining show distinct intracellular patterns among cell types. With the latest lipidomics, mass spectrometry-based lipidomics, lipid imaging, chemical-based lipid analysis and lipid engineering technologies [28,45,48,113,221,222,223], such comparisons can be made, and lipoprotein subtypes can be further classified on the basis of apolipoproteins and lipid differences. In addition, single-cell RNA-sequencing can comprehensively describe cell types and states in human and animal kidneys and identify apolipoprotein expression in each kidney cell, thus revealing CKD-associated molecular mechanisms.

Many kidney diseases occur together with inflammation, a process in which apolipoproteins are studied. For example, apoA-IV has been shown to rescue inflammation by inhibiting proinflammatory cytokine expression, degrading inflammasomes and preventing ROS production [116]. Moreover, increases in lipid accumulation in the kidneys in ApoE-deficient mice are associated with enhanced T cell activation and antigen presentation by dendritic cells [224]. Endogenous apoA-I, apoA-IV and apoE prevent inflammation and oxidative stress from free cholesterol-induced cytotoxicity [225,226,227]. Therefore, consideration must be taken when raising the fact apolipoprotein dysfunction is associated with inflammation levels in CKD. Thus, macrophage lipid metabolism in the kidneys may also play an important role in the pathogenesis of CKD.

Treatment strategies targeting apolipoproteins in CKD have shown varying degrees of success. Methods to specifically target lipoproteins genetically or pharmacologically remain poorly understood, and further knowledge is needed regarding the regulation of specific apolipoproteins and the interplay between CKD and lipoproteins at different cellular signaling levels as well as in different cell types. Nonetheless, current evidence highlights that apolipoprotein perturbations underlie kidney pathology in several types of CKD and may be promising new targets in the search for future therapies.

## Figures and Tables

**Table 1:** Apolipoprotein tissue distribution and lipoprotein content.

Apolipoprotein	Lipoprotein	Main Tissue
ApoA-I	HDL, VLDL, CM	Liver, small intestine, kidney, macrophages
ApoA-II	HDL, VLDL, CM,	Liver, stomach, small intestine, tongue, skin
ApoA-IV	HDL, CM,	Intestine, liver, kidney
ApoA-V	HDL, VLDL, CM,	Liver
ApoB 48	CM, IDL/CM remnants	Intestine,
ApoB100	Lp(a), IDL, LDL, VLDL	Liver
ApoC-I	HDL, IDL, VLDL, CM	Liver, brain
ApoC-II	HDL, IDL, VLDL, CM	Liver, brain
ApoC-III	HDL, IDL/CM remnants, VLDL, CM	Liver, intestine
ApoD	HDL	Brain, kidney, muscle
ApoE	HDL, IDL/CM remnants, VLDL, CM	Liver, kidney, lung, skin
ApoF	HDL, LDL	Liver, kidney, brain
ApoH	HDL	Liver, kidney, lung
ApoJ	HDL	Brain, liver, kidney, lung,
ApoL-1	HDL, LDL	Liver, pancreas, kidney, brain,
ApoL-2	HDL	Liver, kidney, lung, brain
ApoM	HDL, LDL, VLDL, CM	Intestine, liver, kidney

**Table 2:** The physiology and pathophysiology roles of apolipoproteins in the kidneys.

Apolipoprotein	Physiological Functions	Pathophysiology
ApoA-I	main structural protein in HDL, Cholesterol transport	CVD, CKD, FSGS [91,92,93,94,95,97,98]
ApoA-II	main structural protein in HDL, Cholesterol transport	CVD, T2D, DKD, Kidney stones, ESRD [102,103,104,105,106,107]
ApoA-IV	may increase triacylglycerol secretion	DKD, CKD [105,111,115,116,117]
ApoA-V	enhances triacylglycerol uptake	ESRD, CKD, T2D nephropathy [119,120,121,122,123]
ApoB 48	remove excessive triglycerides	albuminuria, gomerulosclerosis, ESRD [94,128,129,130,131,132,133,134]
ApoB100	binds to LDL receptor, remove excessive triglycerides	Albuminuria [132,133]
ApoC-I	activates LCAT	CKD, diabetic nephropathy, glomerulosclerosis, renal cancer [142,147,148,149]
ApoC-II	activates lipoprotein lipase	poorly defined dieases
ApoC-III	inhibits lipoprotein lipase, controls triacylglycerol turnover	renal insufficiency in T2D, kidney stone formation [59,153]
ApoD	associated with LCAT, progesterone binding	poorly defined dieases
ApoE	binds to LDL receptor, remove excessive triglycerides	nondiabetic ESRD, nephrotic syndrome, diabetic nephropathy, ESRD [162,163,164,165,166]
ApoF	inhibits CETP activity	uremia [170]
ApoH	binding to phospholipids	antiphospholipid Syndrome-related kidney dieases [173,174]
ApoJ	cholesterol clearance	acute and chronic renal disease, polycystic kidney disease, ischemic renal tissues, lupus-like nephritis [179,180,181,182,183]
ApoL-1	encodes a secreted HDL, bind to ApoA1, efflux of cholesterol	Renal failure, FSGS, Glomerulonephritis and HIV-related KD [186,187,190,191,192,193]
ApoM	main structural protein in HDL, transports S1P	CKD [202,203]

CETP: cholesteryl ester transfer protein; CKD: chronic kidney disease; CVD: cardiovascular disease; DKD: diabetes kidney disease; ESRD: end stage renal disease; FSGS:focal segmental glomerulosclerosis; HDL: high density lipoprotein; KD: kidney disease; LCAT: Lecithin-Cholesterol Acyltransferase; LDL: low density lipoprotein; S1P:sphingosine-1-phosphate; T2D: type 2 diabetes.
